# Post-traumatic benign paroxysmal positional vertigo: mechanisms, clinical phenotypes, and a structured clinical pathway for management

**DOI:** 10.3389/fneur.2026.1784282

**Published:** 2026-02-03

**Authors:** Tae Hoon Kong, Young Joon Seo

**Affiliations:** 1 Department of Otorhinolaryngology–Head and Neck Surgery, Yonsei University Wonju College of Medicine, Wonju, Republic of Korea; 2 Research Institute of Hearing Enhancement, Yonsei University Wonju College of Medicine, Wonju, Republic of Korea

**Keywords:** post-traumatic benign paroxysmal positional vertigo, benign paroxysmal positional vertigo, canalith repositioning maneuver, head trauma, clinical pathway

## Abstract

Post-traumatic benign paroxysmal positional vertigo (BPPV) is a common but frequently underrecognized cause of dizziness following trauma. Unlike idiopathic BPPV, trauma-related BPPV arises from diverse injury mechanisms and is often characterized by heterogeneous canal involvement, greater need for repeated treatment, and frequent coexistence with broader vestibular dysfunction. These features contribute to diagnostic delays and variable clinical outcomes, particularly in the trauma and emergency care settings. We conducted a structured literature search and synthesized clinical, epidemiological, mechanistic, and implementation-focused evidence across diverse trauma contexts. This review aimed to synthesize current evidence on the mechanisms, epidemiology, and clinical characteristics of post-traumatic BPPV, contrast it with idiopathic BPPV, and propose a pragmatic clinical pathway to improve early recognition and management. We reviewed the clinical, epidemiological, mechanistic, and implementation-focused literature on post-traumatic BPPV across trauma contexts, including head injury, concussion, whiplash, sports-related injury, and traumatic brain injury. Evidence from cohort studies, comparative analyses, meta-analyses, and qualitative and feasibility studies was integrated to inform a clinically oriented framework. Accumulating evidence suggests that post-traumatic BPPV should not be regarded solely as a mechanical disorder of displaced otoconia. Trauma may disrupt the otolithic membrane, promote otoconial detachment, and induce utricular dysfunction, leading to canalithiasis or cupulolithiasis and potential interaction with central vestibular injury. Compared with idiopathic BPPV, post-traumatic cases more frequently involve horizontal or multiple canals, often require repeated canalith repositioning maneuvers, and demonstrate variable recurrence patterns. System-level barriers, including limited screening, insufficient training, and fragmented care pathways, further contribute to underdiagnosis and suboptimal management. Post-traumatic BPPV represents a distinct clinical phenotype within the spectrum of trauma-related vestibular disorders. Early identification through systematic screening, comprehensive positional testing, and timely canal-specific interventions provides practical opportunities to improve outcomes. We propose a structured clinical pathway emphasizing early recognition, planned reassessment, and escalation to integrated vestibular care when symptoms persist. Future research should clarify the relationships between trauma biomechanics and BPPV phenotypes, identify predictors of recurrence, and evaluate the real-world effectiveness of pathway-based care models across diverse trauma populations.

## 1 Introduction

Benign paroxysmal positional vertigo (BPPV) is the most common cause of peripheral vertigo and has traditionally been regarded as an idiopathic condition resulting from the displacement of otoconia into the semicircular canals (1). However, a substantial proportion of BPPV cases are secondary to identifiable conditions, with trauma consistently recognized as the leading cause of secondary BPPV (2). Population-based and clinical cohort studies indicate that the risk of developing BPPV increases after head trauma compared with the general population, even after injuries that are clinically categorized as mild (3). Investigations of trauma-specific risk factors further highlight the need to consider BPPV proactively rather than incidentally in affected patients (4).

Post-traumatic BPPV encompasses a wide spectrum of injury mechanisms. It has been described after direct head impact and mild head trauma (5), as well as after sports-related concussions in adolescents and young adults (6) and in pediatric concussion (7). BPPV has also been reported following whiplash injuries without direct head impact (8) and chronic symptom complexes, including whiplash-associated disorders, have been discussed in relation to chronic or recurrent BPPV phenotypes (9) In addition, blast- or vibration-related exposures have been implicated in cases attributed to cannon fire and similar sources (10). More recently, emerging or unusual triggers have been proposed in case-based literature, such as “earbuds-induced” BPPV, illustrating both the breadth of hypothesized mechanisms and the need for careful clinical adjudication (11).

Clinically, post-traumatic BPPV may be overlooked or misdiagnosed, particularly in acute trauma or emergency settings, where dizziness is often attributed to central injury or post-concussive syndromes. Comprehensive reviews of post-concussive dizziness emphasize the heterogeneity of vestibular and non-vestibular contributors, which can obscure treatable peripheral diagnoses such as BPPV (12). Studies of vestibular dysfunction in acute traumatic brain injury (TBI) further demonstrate that vestibular abnormalities are common early after injury, reinforcing the need for targeted bedside evaluation (13).

Accumulating evidence suggests that post-traumatic BPPV may differ from idiopathic BPPV in clinically meaningful ways, including canal distribution, treatment response, and recurrence patterns. Several comparative studies have directly contrasted post-traumatic and idiopathic phenotypes (1, 14, 15), and more recent work has examined these differences using a retrospective design (16). A meta-analysis has synthesized evidence on treatment response and recurrence differences between traumatic and idiopathic BPPV, illustrating both shared features and substantial heterogeneity across studies (17).

Finally, practical barriers to diagnosis and treatment in trauma settings are increasingly recognized. Mixed-methods and qualitative investigations have described why patients with acute TBI are not routinely assessed or treated for vestibular dysfunction and have explored the perspectives in trials of acute post-traumatic BPPV management (18–20). These implementation gaps render post-traumatic BPPV a particularly suitable subject for a clinically oriented review incorporating a pragmatic care pathway proposal.

In this review, we summarize the current evidence regarding the mechanisms, epidemiology, and clinical characteristics of post-traumatic BPPV; contrast these features with idiopathic BPPV; and propose a practical clinical pathway emphasizing early recognition, structured assessment, and appropriate follow-up.

## 2 Methods: literature search strategy and study selection

We conducted a structured literature search to identify studies relevant to post-traumatic benign paroxysmal positional vertigo (BPPV). PubMed/MEDLINE and Embase were searched from inception to December 2025 using combinations of keywords and controlled vocabulary terms including “benign paroxysmal positional vertigo,” “BPPV,” “post-traumatic,” “head trauma,” “concussion,” “whiplash,” and “traumatic brain injury.” Eligible articles included human clinical studies addressing mechanisms, epidemiology, clinical phenotypes, canal involvement patterns, treatment response, recurrence, or care delivery and implementation issues in trauma-related BPPV. Systematic reviews and meta-analyses were included when available. Case reports were selectively included only when they provided unique insights into rare phenotypes (e.g., bilateral or multicanal disease) or uncommon trauma contexts (e.g., blast or vibration exposure). Studies focusing solely on non-positional dizziness without evidence of BPPV were excluded. The final reference list was curated to provide a clinically oriented synthesis across diverse trauma settings.

## 3 Mechanisms of post-traumatic BPPV

### 3.1 Trauma-induced otoconial detachment and canalithiasis/cupulolithiasis

The most widely accepted mechanism of post-traumatic BPPV is trauma-induced detachment of otoconia from the otolithic membrane—most commonly the utricular macula—followed by migration into the semicircular canals, resulting in canalithiasis or, less frequently, cupulolithiasis (1). A survey examining the nature of trauma in post-traumatic BPPV supports the concept that diverse traumatic forces may precede symptom onset, consistent with a mechanical trigger for otoconial displacement (21). Importantly, the occurrence of BPPV following mild head trauma suggests that substantial intracranial injury is not required to destabilize otoconia and that relatively modest biomechanical insults may be sufficient to initiate this process (3, 5).

### 3.2 Whiplash and non-impact mechanisms

A distinctive feature of post-traumatic BPPV is that it may occur after acceleration–deceleration injuries without direct head impact. Whiplash-associated BPPV has been explicitly examined, raising the possibility that rapid rotational or torsional forces transmitted to the labyrinth can precipitate otoconial detachment (8). In broader clinical discussions, chronic BPPV in individuals with a history of trauma has been considered in relation to symptom triggers and overlapping syndromes, including whiplash-associated disorders (9). Although causality and directionality remain complex, these reports collectively support a mechanism in which inertial forces, rather than impacts per se, destabilize otolith structures.

### 3.3 Vibration, blast, and other unusual exposures

Post-traumatic BPPV after intense vibrations or blast-like exposure has also been reported. A case report attributed the onset of BPPV to cannon fire, suggesting that high-energy acoustic or vibrational forces may contribute to otoconial instability in susceptible individuals (10). Similarly, recent discussion of possible “earbuds-induced” BPPV—although not traumatic in the conventional sense—highlights ongoing exploration of how repeated vibration or mechanical stimulation might relate to positional vertigo, while emphasizing the importance of cautious interpretation and differential diagnosis (11). Dental procedures have also been proposed as potential triggers for BPPV, possibly due to sustained head positioning and exposure to high-frequency vibrational stimuli during treatment, which may facilitate otoconial detachment in susceptible individuals (16). These observations broaden the proposed mechanistic framework while underscoring that evidence quality varies, and that mechanistic claims should remain proportionate to study design.

### 3.4 Otolith dysfunction and recurrence susceptibility

Beyond the initial mechanical insult, trauma may induce more diffuse otolith organ dysfunction, potentially increasing susceptibility to recurrence. Otolith dysfunction in recurrent BPPV following mild TBI has been demonstrated using vestibular-evoked myogenic potentials, supporting the concept that utricular impairment may contribute to recurrent or refractory disease (22). This interpretation aligns with clinical observations that post-traumatic BPPV often requires repeated canalith repositioning maneuvers and may recur unpredictably (16, 17).

### 3.5 TBI as a setting for combined peripheral and central vestibular injury

Vestibular symptoms following TBI often reflect a combination of peripheral and central injuries. Studies on acute TBI have documented vestibular dysfunction and have highlighted that vestibular end-organ injury may coexist with impairment of central vestibular pathways (13). In this context, BPPV may represent only one component of a broader dizziness syndrome, which can influence symptom persistence and complicate assessment of treatment response after repositioning maneuvers (12, 23).

**Figure 1:**
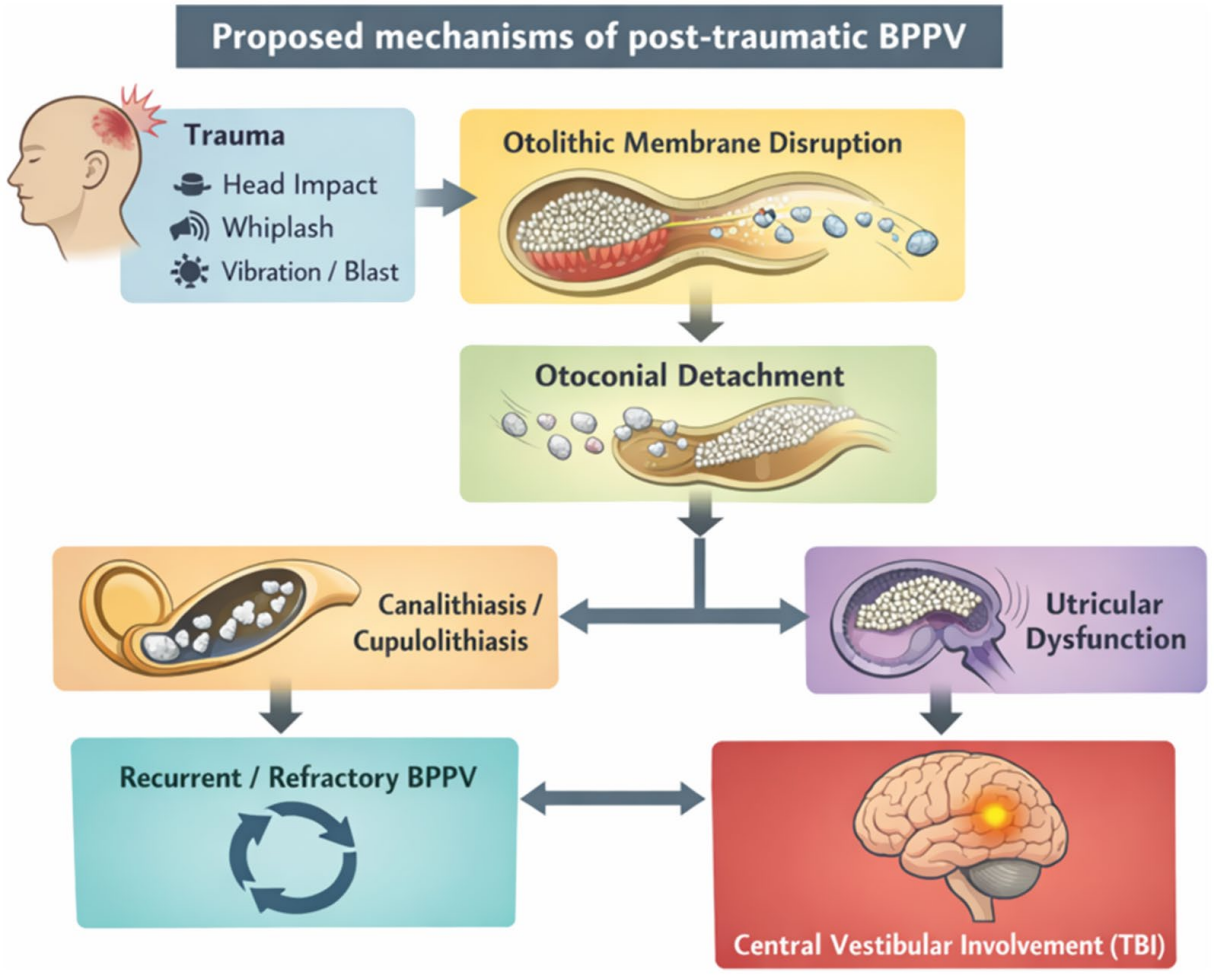
Proposed mechanisms of post-traumatic benign paroxysmal positional vertigo.

Taken together, accumulating evidence suggests that post-traumatic BPPV should not be conceptualized solely as a mechanical disorder caused by displaced otoconia. Rather, trauma may disrupt the otolithic membrane, promote otoconial detachment, and induce utricular dysfunction, with downstream development of canalithiasis or cupulolithiasis and potential interaction with central vestibular injury in the setting of TBI (Figure 1).

## 4 Epidemiology and trauma phenotypes

### 4.1 Risk and frequency across settings

The risk of developing BPPV after head trauma has been quantified in cohort studies, demonstrating an increased incidence compared with non-trauma populations (3). In a prospective study of 117 adults with minimal-to-moderate head injury, 21% developed definite traumatic BPPV within 3 months (95% CI, 14%–29%), with most cases occurring within the first 2 weeks after trauma (3). Within the broader landscape of secondary BPPV, multicenter data underscore the clinical importance of identifiable causes and frame trauma-related BPPV as a major, potentially actionable subset (2). In a nationwide multicenter study, secondary BPPV accounted for 14.5% of all BPPV cases, and head trauma was the most common presumed etiology (2). Risk factor analyses performed within trauma cohorts may further inform future screening strategies; in a Korean trauma database study, BPPV prevalence was approximately 1.0%, and head-and-neck injury was a significant risk factor (OR 10.556, 95% CI 1.029–108.262) (4).

### 4.2 Mild head trauma, concussion, and sports-related phenotypes

The trauma phenotypes associated with post-traumatic BPPV are summarized in Table 1. A recurring phenotype is BPPV following mild traumatic injury, including concussion. Adolescents and young adults with recent concussions have been studied, with reports describing clinical outcomes across BPPV diagnoses in these populations (6). Similarly, pediatric sports-related concussion cohorts include documented cases of post-concussion BPPV, reinforcing that traumatic BPPV is not confined to older adults (7). In sports contexts, BPPV has been reported in adolescent rugby players (24), and its evaluation has been described in American football players (25). Soccer-related cases have also been reported, illustrating that even noncollisional repetitive head impacts or sports exposure may be relevant in some individuals (26). Collectively, these studies support the practical implication that positional testing should be considered during the evaluation of post-concussion dizziness across age groups.

**Table 1:** Trauma phenotypes associated with post-traumatic benign paroxysmal positional vertigo.

Trauma phenotype	Representative context	Characteristic features	Key references
Mild head trauma	Falls, minor accidents	BPPV without imaging abnormalities	Gordon et al. (1); Balatsouras et al. (5)
Concussion/mild TBI	Sports-related concussion	Often under-recognized; may require repeated CRM	Bowman et al. (6); Kim et al. (23)
Moderate–severe TBI	High-energy trauma	Coexisting central vestibular deficits	Marcus et al. (13)
Whiplash injury	Acceleration–deceleration injury	BPPV without direct head impact	Dispenza et al. (8)
Sports-related trauma	Rugby, football, soccer	Horizontal canal or multi-canal BPPV	Bashir et al. (24); Warming et al. (26)
Pediatric trauma	Youth sports concussion	Delayed diagnosis common	Reimer et al. (7)
Craniofacial trauma	Mandibular fracture	Mechanical force transmission to the labyrinth	Bashir et al. (27)
Blast/vibration exposure	Cannon fire, blast	Rare, but illustrative of unusual triggers	Kauffman (10)
Complex/refractory	Bilateral or multi-canal involvement	Treatment-resistant	Maslovara et al. (28); Dundar et al. (29)

BPPV, benign paroxysmal positional vertigo; TBI, traumatic brain injury; CRM, canalith repositioning maneuvers.

### 4.3 Whiplash-associated and neck-injury phenotypes

Whiplash-associated BPPV remains a debated phenotype but has been repeatedly described, including studies that directly question whether BPPV following whiplash injury represents a myth or a clinical reality (8). In some clinical discussions, chronic BPPV in individuals with a history of trauma has been considered in relation to symptom triggers within broader syndromic contexts, including whiplash-associated disorders (9). Although the strength of evidence varies, these reports collectively justify including whiplash and neck injuries as clinical triggers for screening for positional vertigo.

### 4.4 Blast, vibration, and rare trauma mechanisms

Beyond conventional trauma mechanisms, the case-based literature describes unusual exposures, such as cannon fire-related traumatic BPPV (10). Mandibular fracture–associated BPPV has also been reported, emphasizing that craniofacial trauma and transmitted forces may be relevant, even when the primary injury is not intracranial (27). Although such reports are not suitable for prevalence estimation, they are valuable for broadening clinical awareness of possible phenotypes and diagnostic considerations.

### 4.5 Canal involvement patterns and atypical presentations

Trauma-related BPPV is frequently characterized by greater heterogeneity in canal involvement, with increased reports of horizontal canal involvement and multi-canal disease compared with idiopathic BPPV (14, 15). Rare presentations, such as bilateral post-traumatic BPPV, have been published (28), and refractory multicanal BPPV has also been described in case reports (29). Additionally, trauma-induced unilateral horizontal canal BPPV with apogeotropic nystagmus has been documented in adolescents, underscoring the need to consider less common variants in younger patients with trauma (30). Finally, the relationship between focal TBI location and canal involvement has been explored, suggesting that injury distribution may influence clinical patterns; however, further validation is required (31).

### 4.6 Age-specific considerations: pediatric and older adults

Post-traumatic BPPV has important age-specific implications. In children and adolescents, trauma is frequently reported as a major precipitating factor for BPPV, particularly in the context of sports-related concussion, and delayed recognition may prolong symptoms and interfere with return-to-school or return-to-play protocols (23, 32, 33). In older adults, post-traumatic BPPV is clinically relevant not only because falls may precipitate otoconial detachment, but also because untreated BPPV itself increases the risk of recurrent falls and related morbidity (25, 26). Therefore, positional testing should be considered systematically in older adults presenting after a fall or minor head injury, especially when dizziness, imbalance, or motion-provoked symptoms are reported (25, 26).

## 5 Clinical characteristics compared with idiopathic BPPV

Evidence increasingly supports clinically meaningful differences between post-traumatic and idiopathic BPPV, with implications for diagnostic evaluation, treatment planning, and follow-up strategies. The key clinical distinctions between post-traumatic and idiopathic BPPV are summarized in Table 2.

**Table 2:** Comparison between post-traumatic and idiopathic benign paroxysmal positional vertigo.

Feature	Post-traumatic BPPV	Idiopathic BPPV
Typical etiology	Head injury, concussion, whiplash, craniofacial trauma, or sports-related injury	Age-related otolithic degeneration
Affected population	All ages, including pediatric and young adults	Predominantly middle-aged and older adults
Time to symptom onset	Immediate or delayed (days to months)	Usually spontaneous
Underlying mechanism	Trauma-induced otoconial detachment ± utricular dysfunction	Degenerative otoconial detachment
Canal involvement	Higher frequency of horizontal canal, bilateral, or multi-canal disease	Mostly posterior canal
Atypical presentations	Frequent (bilateral, refractory, apogeotropic)	Uncommon
Associated vestibular deficits	Common, especially in TBI	Rare
Response to CRM	Often requires repeated sessions	Typically resolves with 1–2 sessions
Recurrence pattern	Variable and sometimes unpredictable	Relatively predictable
Management focus	Early screening, structured follow-up, and integrated care	Standard repositioning maneuvers

BPPV, benign paroxysmal positional vertigo; TBI, traumatic brain injury; CRM, canalith repositioning maneuvers.

### 5.1 Canal distribution and complexity

Compared with idiopathic BPPV, post-traumatic cases appear more likely to involve the horizontal canals, multiple canals, or bilateral disease across several cohorts (14, 15). Such distributions may produce more complex nystagmus patterns and often require comprehensive testing beyond a single Dix–Hallpike maneuver. Comparative analyses of traumatic versus idiopathic BPPV have emphasized differences in disease characteristics and outcomes (14), and personal-experience series have similarly highlighted the need to anticipate atypical or multicanal involvement in post-traumatic BPPV (15). Case-based literature further underscores that bilateral involvement can occur following trauma (28) and that refractory multicanal disease may pose substantial clinical challenges (29).

### 5.2 Treatment response and the need for repeated maneuvers

Multiple studies have indicated that post-traumatic BPPV may require a greater number of repositioning maneuvers to achieve symptom resolution than idiopathic BPPV. Comparative analyses of symptom resolution rates between post-traumatic and non-traumatic BPPV support clinically relevant differences in recovery trajectories (32). More recently, a retrospective comparison of post-traumatic and idiopathic BPPV identified differences in clinical characteristics, aligning with the concept that trauma-related BPPV may follow a more treatment-intensive course (16). Additional analyses of disease, treatment, and outcome characteristics further suggest that traumatic BPPV may present features relevant to management planning (14). A meta-analysis comparing treatment and recurrence outcomes between traumatic and idiopathic BPPV provided a broader synthesis and demonstrated heterogeneity across studies (17).

### 5.3 Recurrence: variability and proposed contributors

Recurrence patterns in post-traumatic BPPV vary across studies and may depend on injury phenotype, canal involvement, duration of follow-up, and the presence of concomitant vestibular damage. Early studies questioned whether post-traumatic BPPV differs meaningfully from idiopathic BPPV, highlighting the need for careful comparative interpretation (1). Meta-analytic synthesis has demonstrated variability in recurrence outcomes, reinforcing that “one-size-fits-all” assumptions regarding recurrence are inappropriate (17). Otolith dysfunction, particularly utricular impairment, has been implicated in recurrent BPPV after mild TBI, suggesting a plausible biological link between trauma and recurrence susceptibility (22).

### 5.4 Comorbidity, underlying medical conditions, and broader dizziness syndromes

Trauma-related dizziness often coexists with other vestibular and neurological symptoms. Reviews of post-concussive dizziness emphasize that persistent dizziness may reflect multiple underlying mechanisms and that treatable peripheral causes, such as BPPV, should not be overlooked (12). In addition, differences in the clinical course of BPPV according to underlying medical conditions have been reported, suggesting that host factors and comorbidities may modulate symptom trajectories and recurrence risk across BPPV phenotypes (33). Some hypotheses have linked chronic trauma-related BPPV with symptom triggers in vestibular migraines or other syndromic contexts; however, these relationships require careful interpretation and should not substitute for standard diagnostic criteria (9).

## 6 Post-traumatic BPPV in traumatic brain injury

TBI represents a clinical context in which post-traumatic BPPV is both relevant and frequently underrecognized. Vestibular dysfunction is prevalent in the acute phase of TBI, and associated symptoms may reflect a combination of peripheral and central pathologies (13). Because dizziness is often attributed to central mechanisms or nonspecific post-concussive syndromes, systematic positional testing may be omitted, thereby delaying the diagnosis and treatment of BPPV (12, 19).

Clinical studies have examined BPPV after TBI and have described characteristic features of this population. For example, BPPV following TBI has been characterized in clinical cohorts, emphasizing that TBI-associated dizziness may include treatable positional vertigo (23). This observation aligns with broader evidence that mild head trauma can precipitate BPPV and that clinical vigilance should not be reserved exclusively for severe injuries (3, 5).

A key clinical challenge is that BPPV often does not occur in isolation in the setting of TBI. Vestibular dysfunction in acute TBI may include oculomotor abnormalities, balance impairment, and central processing disturbances, such that symptom persistence after successful repositioning may be misinterpreted as “failed” BPPV treatment rather than residual non-BPPV vestibular pathology (12, 13). This consideration underscores the importance of integrated assessment and longitudinal follow-up rather than single-episode, maneuver-centric care.

## 7 Management considerations: what should be different?

Although canalith repositioning maneuvers remain the cornerstone of treatment, the management of post-traumatic BPPV differs from that of idiopathic BPPV in several practical aspects, including the need for early recognition in non-otologic settings, anticipation of repeated treatment sessions, attention to coexisting vestibular deficits, and structured follow-up.

### 7.1 Early recognition and intervention in trauma settings

Evidence supports the clinical value of early identification and management of BPPV in trauma populations. Early management of post-traumatic BPPV has been investigated in injured patients, demonstrating that prompt assessment and treatment are feasible and may improve recovery trajectories (34). Similarly, feasibility trials indicate that structured approaches to BPPV management in acute TBI settings are possible and can be studied systematically (18). Nevertheless, qualitative research has highlighted that patients with acute TBI are not routinely assessed or treated for vestibular dysfunction in some healthcare systems because of barriers such as unclear clinical responsibility, time constraints, and limited provider training (19).

### 7.2 Counseling and expectation management: repeated maneuvers and variable recovery

As post-traumatic BPPV often requires repeated repositioning sessions, clinicians should explicitly counsel patients that symptom resolution may require multiple treatments and that scheduled reassessment is appropriate (14, 16, 32). A meta-analytic synthesis supports the conclusion that outcomes and recurrence are more variable in traumatic BPPV than in idiopathic BPPV, reinforcing the need for individualized counseling and follow-up planning (17).

### 7.3 Integrated care for persistent symptoms

Persistent dizziness after successful canalith repositioning is common in the context of trauma and may reflect coexisting vestibular dysfunction rather than persistent BPPV. Reviews of post-concussive dizziness emphasize that clinicians should evaluate multiple contributing mechanisms and may need to implement vestibular rehabilitation strategies alongside canalith repositioning maneuvers (12). Data from acute TBI populations further support the observation that vestibular dysfunction is prevalent and not limited to canalithiasis, making integrated vestibular care clinically appropriate (13). Where available, targeted vestibular rehabilitation can address residual imbalance, motion sensitivity, and oculomotor deficits after BPPV resolution.

### 7.4 System-level barriers and implementation priorities

Implementation barriers are not purely clinical; they are organizational in nature. Qualitative studies have described why vestibular assessment is not routinely incorporated into acute TBI care and how both patients and clinicians perceive trial participation and proposed care pathways (19, 20). These data suggest several practical interventions, including focused training for non-otologic clinicians (such as emergency physicians, trauma teams, and rehabilitation providers), the use of simple screening triggers, and the establishment of clear referral pathways. In addition, trial feasibility studies provide a foundation for pathway development and iterative refinement (18).

### 7.5 Why post-traumatic BPPV may be more refractory to repositioning maneuvers

Although the exact reasons remain incompletely understood, several factors may explain why post-traumatic BPPV often requires repeated canalith repositioning maneuvers and may appear more difficult to treat than idiopathic BPPV (22). First, traumatic cases more frequently involve horizontal canal, multicanal, or bilateral disease, which increases diagnostic complexity and may require sequential or repeated maneuvers (19, 20). Second, trauma may induce broader otolith organ dysfunction (particularly utricular impairment), potentially increasing the likelihood of recurrent otoconial detachment and symptom relapse even after initially successful treatment (15, 16). Third, in patients with concussion or traumatic brain injury, persistent dizziness after repositioning may reflect coexisting central vestibular dysfunction, oculomotor abnormalities, or balance impairment rather than ongoing BPPV, which can be misinterpreted as treatment failure (13, 32, 33). Finally, associated cervical pain or limited range of motion after head–neck trauma may restrict optimal positioning during maneuvers and delay symptom resolution (12, 31). These considerations support planned reassessment and integrated vestibular care in patients with persistent symptoms.

## 8 Clinical pathway proposal: a practical approach to post-traumatic BPPV

**Figure 2:**
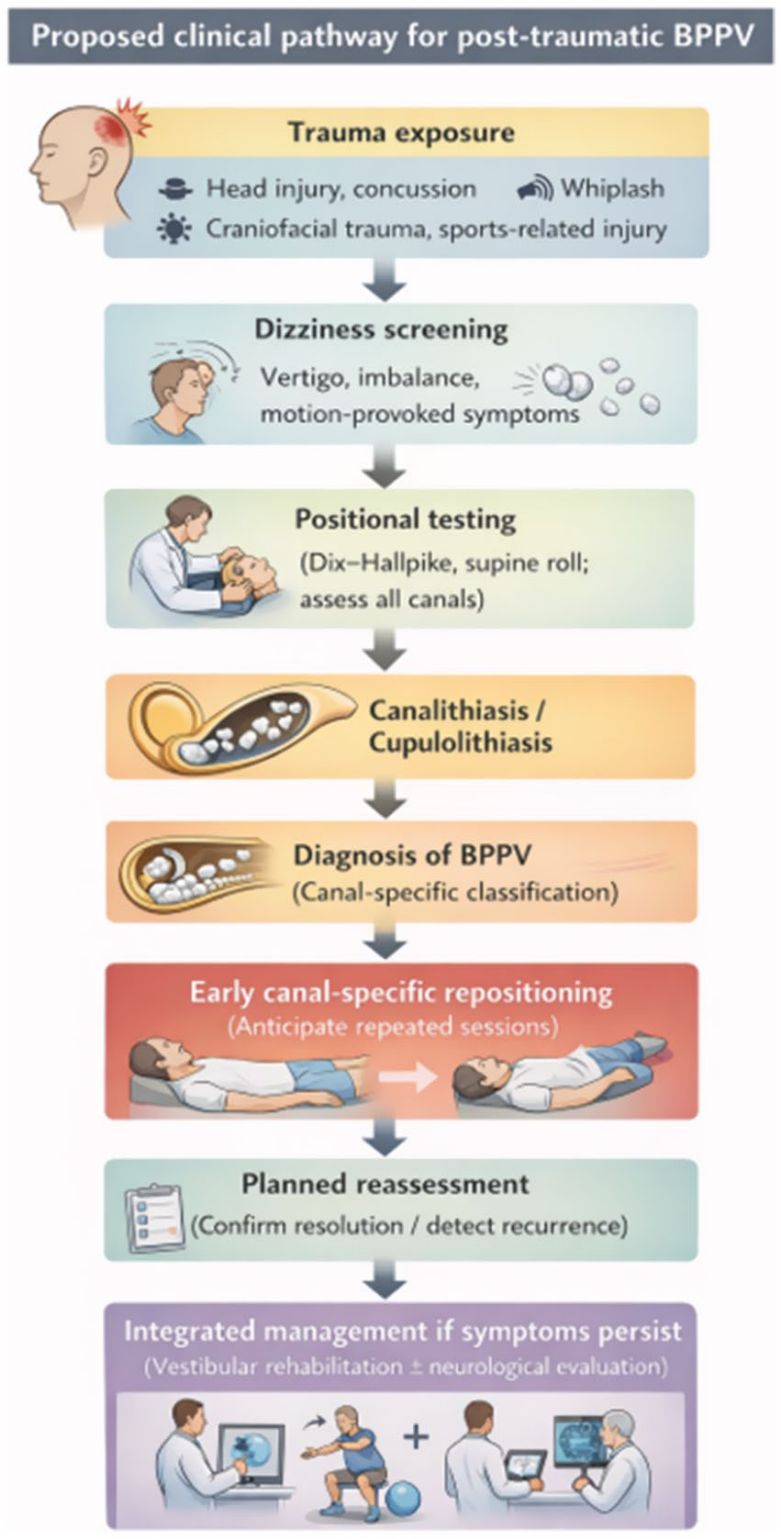
Proposed clinical pathway for post-traumatic benign paroxysmal positional vertigo.

Considering the predictable diagnostic gaps and management challenges in trauma settings, we propose a structured clinical pathway that emphasizes early screening, comprehensive positional testing, timely canal-specific treatment, planned reassessment, and escalation to integrated vestibular care when symptoms persist (Figure 2).

### 8.1 Step 1: screening trigger (who should be tested?)

Any trauma patient reporting vertigo, dizziness, imbalance, or motion-provoked symptoms should be considered for positional testing, regardless of injury severity or imaging findings (3, 23). Screening should include not only direct head impact but also whiplash mechanisms and craniofacial injuries, consistent with reports of whiplash-related and mandibular fracture–associated BPPV (8, 27). In adolescent and sports-concussion populations, vestibular screening should be routine, as BPPV has been reported in these settings and may be readily treatable within a broader post-concussion care plan (6, 7, 24).

### 8.2 Step 2: standardized positional testing (test all relevant canals)

Because trauma-related BPPV more frequently involves horizontal canals and multi-canal patterns, positional testing should be comprehensive and include both the Dix–Hallpike and supine roll tests, rather than being limited to suspected posterior canal disease (14, 15). Clinicians should remain alert to less common variants, such as apogeotropic horizontal canal BPPV, which has been described in trauma-related adolescent cases (30). Careful documentation and repeat testing are essential in suspected bilateral or refractory cases, as bilateral BPPV and refractory multicanal presentations have been reported following trauma (28, 29).

### 8.3 Step 3: early canal-specific treatment and planned reassessment

Once diagnosed, canal-specific repositioning maneuvers should be performed as early as feasible. Evidence supports the early management of traumatically injured patients, and feasibility trial data further support pathway-based intervention approaches in acute TBI care (18, 34). Given the higher likelihood of requiring repeated sessions in post-traumatic BPPV, the pathway should incorporate planned reassessment rather than relying solely on symptom-driven return visits (16, 32). Meta-analytical evidence indicates heterogeneity in recurrence rates and treatment response, reinforcing the need for structured follow-up (17).

### 8.4 Step 4: evaluate persistent symptoms and refer for integrated care when needed

If dizziness persists after successful BPPV treatment, the pathway should prompt assessment for coexisting peripheral vestibular or central contributors and consideration of vestibular rehabilitation (12, 13). The high prevalence of vestibular dysfunction in TBI cohorts supports integrating BPPV treatment within a broader, multidisciplinary dizziness care framework (13, 23). When recurrence is suspected, clinicians may consider the adjunctive evaluation of otolith function, acknowledging the evidence of utricular dysfunction in recurrent BPPV following mild TBI (22).

### 8.5 Step 5: implementation and patient-centered outcomes

Pathway implementation should explicitly address barriers identified in qualitative studies, such as unclear professional responsibility, limited training, and time constraints inherent to acute care settings (19). Patient perspectives derived from trial participation suggest that early diagnosis and treatment may reduce anxiety and improve confidence in recovery, supporting the value of pathway-based care beyond symptom resolution alone (20). In sports contexts, incorporating positional testing into post-concussion protocols can reduce prolonged symptoms in adolescents and young adults (6, 7). Finally, unusual triggers reported in the literature, such as blast exposure or vibration-related hypotheses, should encourage diagnostic openness while maintaining a rigorous differential diagnosis (10, 11).

## 9 Knowledge gaps and future directions

Despite the growing literature, several knowledge gaps continue to limit the delivery of precision care for post-traumatic BPPV.

### 9.1 Linking injury biomechanics to phenotype

It remains unclear how trauma type, direction, and severity influence canal involvement, bilaterality, and treatment response. Existing studies suggest that focal TBI location may be associated with specific canal involvement; however, validation across larger cohorts and diverse injury mechanisms is required (31). Similarly, the relationship between whiplash injury and BPPV remains debated, and prospective studies are needed to clarify causal relationships and risk gradients (8, 9).

### 9.2 Predicting recurrence and refractory disease

Reliable predictors of recurrence and refractory disease are currently lacking. Evidence of utricular dysfunction in recurrent BPPV following mild TBI suggests that otolith testing may help stratify recurrence risk; however, optimal clinical workflows and predictive utility remain to be established (22). A systematic synthesis reported variable recurrence outcomes between traumatic and idiopathic BPPV, highlighting the heterogeneity that future stratified studies should address (17).

### 9.3 Standardizing care pathways and evaluating real-world effectiveness

Feasibility trials provide an initial foundation for pathway-based care in acute TBI settings; however, larger pragmatic studies are needed to evaluate real-world effectiveness, sustainability, and cost implications (18). Qualitative research demonstrates persistent barriers to vestibular assessment and treatment in trauma settings, and implementation studies should test targeted training models and role-delineation strategies to address these challenges (19, 20). Early management strategies in traumatically injured patients should also be extended across diverse health systems and populations, including sports and pediatric settings (6, 7, 34).

### 9.4 Expanding phenotype characterization across age groups and triggers

Pediatric and adolescent phenotypes warrant dedicated studies, as sports-related concussion cohorts demonstrate the occurrence of BPPV; however, standardized screening approaches remain inconsistent across settings (6, 7). Evaluations and case reports from contact sports, including rugby, American football, and soccer, highlight the need for consistent clinical algorithms (24–26). Finally, emerging hypotheses regarding vibration-related triggers (e.g., blast exposure and so-called “earbuds-induced” cases) raise questions that require careful epidemiologic and mechanistic investigation to distinguish true associations from coincidental temporal relationships (10, 11).

## 10 Conclusion

Post-traumatic BPPV is a common yet frequently under-recognized cause of dizziness following trauma. Compared with idiopathic BPPV, trauma-related cases arise from diverse mechanisms, including head impact, concussion, whiplash, craniofacial injuries, and unusual vibrational exposures, and may exhibit greater heterogeneity in canal involvement and clinical course (8, 10, 16, 21). Evidence suggests that post-traumatic BPPV more often requires repeated repositioning maneuvers and may present with variable recurrence patterns, possibly influenced by broader otolith dysfunction and multisystem vestibular injury (13, 17, 22, 32).

Because post-traumatic dizziness is frequently attributed to central or nonspecific post-concussive mechanisms, systematic positional testing is essential for identifying BPPV as a treatable contributor, including in mild injuries and pediatric/sports contexts (6, 7, 12, 23). Early assessment and canal-specific treatment, supported by structured follow-up and integrated management of persistent symptoms, represent practical opportunities to improve patient outcomes (18, 34).

A pragmatic clinical pathway—including screening, comprehensive positional testing, early intervention, planned reassessment, and escalation to integrated vestibular care when needed—can address predictable diagnostic gaps and standardize care across emergency, trauma, and rehabilitation settings (19, 20). Future studies should clarify how trauma biomechanics shape BPPV phenotypes, identify objective predictors of recurrence, and evaluate the effectiveness of pathway-based models across diverse populations.
